# Update of Cestodes Parasitizing Neotropical Hystricomorphic Rodent

**DOI:** 10.3389/fvets.2022.885678

**Published:** 2022-04-29

**Authors:** Kegan Romelle Jones

**Affiliations:** ^1^Department of Basic Veterinary Sciences, Faculty of Medical Sciences, School of Veterinary Medicine, University of the West Indies, St. Augustine Campus, Mt. Hope, Trinidad and Tobago; ^2^Department of Food Production, Faculty of Food and Agriculture, University of the West Indies, St. Augustine Campus, St. Augustine, Trinidad and Tobago

**Keywords:** agouti, lappe, capybara, *Dasyprocta leporina*, *Hydrochoerus hydrochaeris*, *Agouti paca*, nutria, vizcacha

## Abstract

This review aims at identifying cestodes that are present in hunted rodent species in the neo-tropical region. The rodent species that was investigated were the capybara (*Hydrochoerus hydrochaeris*, Linnaeus, 1766), lappe (*Cuniculus paca*, Linnaeus, 1766), agouti (*Dasyprocta leporina*, Linnaeus, 1758), chinchilla (*Chinchilla chinchilla*, Lichtenstein, 1829), Trinidad spiny rat (*Proehimys trinitatus*, Allen and Chapman, 1893), nutria (*Myocastor coypus*, Molina, 1782), and vizcacha (*Lagostomus maximus*, Desmarest, 1817). These rodent species are utilized for their meats in many rural communities in the Caribbean and South America. These rodents belong to the hystricomorphic group. *Raillietina demerariensis* Daniels, 1895 was commonly found in the gastrointestinal tract of *D. leporina, C. paca* and *P. trinitatus*. Similarly, in the liver, muscle and subcutaneous tissue the metacestodes on *Echinococcus vogeli* Daniels, 1895 and *Echinococcus oligarthrus* was found in the lappe and agouti. The capybara was found to have the most species of cestodes in its gastrointestinal tract when compared to the agouti and lappe. However, metacestodes were not recorded in the tissues of the capybara. This surprising feature shows the effect of the difference in feeding habits between the capybara and the agouti and lappe. The literature reviewed in this study includes scientific publications on cestodes and metacestodes of Hystricomorphic rodents. An exhaustive search was performed using the digital repositories in Google Scholar, Scielo, Redalyc, Scopus and Pubmed. Literature searched spanned the years 1970-2021. Cestodes of zoonotic significance were *E. vogeli* and *E. oligarthrus*, with humans becoming infected when consuming eggs of contaminated food and water. The agouti and lappe act as intermediate host in the life cycle of *E. vogeli* and *E. oligarthrus*, the definitive host (canids and felids) become infected by consuming of tissue infected with metacestodes. Humans become infected through the ingestion of eggs from the definitive host where cystic lesions develop in the liver, lungs and other abdominal organs.

## Introduction

Hystricomorphic rodents that are present in the neo-tropics have tremendous potential for domestication (1). These rodents are utilized for their meat and hides (2, 3). These animals also have the ability to harbor adult cestodes in their gastrointestinal tract as well as metacestodes in other tissues. These neo-tropical rodents are being reared in captivity as for their meat due to their ability to consume local feed resources (4). These animals can serve as reservoirs for many diseases which can be transmitted to humans (5). The meat of these animals has been found to be very nutritious with high levels of protein and unsaturated fatty acids (6–8). It is through the hunting of these animals that humans may be indirectly infected with cestodes from these animals.

Cestodes which are present in their definitive hosts show little clinical signs. However, in large numbers may lead to impaction and some gastrointestinal disturbances (9). In the intermediate host these cestodes can cause greater harm depending on the organ which is affected. Due to the increased utilization of these rodents either through hunting or wildlife farming the understanding of these cestodes in the digestive system and other organs must be known and highlighted. As such the aim of this review is to highlight the cestodes which are present in the gastrointestinal tract as well as other tissues in neo-tropical hystricomorphic rodents. Metacestodes which are found in other tissues will also be discussed and the potential impact these cestodes have on human health for persons in the neo-tropics.

## Gastrointestinal Cestodes

Tapeworms which are found in the gastrointestinal tract are usually seen in the definitive host (9). As such, parasites discussed in this section have the capybara (*H. hydrochaeris*), agouti (*D. leporina*) and lappe (*C. paca*) as their definitive host. In most cases adult tapeworms usually cause no clinical signs of diseases (10). However, in large numbers adult cestodes may cause impaction, malnutrition and enteritis (10). The agouti and the lappe are medium sized hystricomorphic rodent with similar feeding habits. As such, both animals have been reported to share some species of cestodes (11). The capybara is the largest rodent on this planet and has feeding habits which are very different to its aforementioned counterparts. *Raillietina demerariensis* has been reported in the Trinidad spiny rat, agouti and lappe (11–13).

Suepaul et al. (14) identified the cestodes ova in a single sample but the adult forms could not be found in the intestinal content of the agouti. Eggs of *Hymenolepsis diminuta* Ransom, 1901and *Taenia* spp. Goeze, 1782 were noted in captive reared lappe (15, 16). However, the prevalence of these cestodes in the agouti and lappe were quite small (see Table 1). In contrast, there have been several studies investigating gastrointestinal parasites in the capybara. Most studies have identified six species of cestodes in the capybara with a higher prevalence than those reported in the lappe and agouti (see Table 1). Cestodes frequently reported included: *Monoecocestus hagmanni* Beddard, 1914, *Monoecocestus hydrochoerus, Monoecocestus* spp., *Monoecocestus jacobi, Monoecocestus macrobursatum* and Anoplocephalidae (17–27). It should be noted that in most cases cestodes found in the gastrointestinal tract did not result in clinical signs of diseases (9), however, Salas and Herrera (25) noted that there was a negative association between capybara infected with *H. macrobursatum* and their body condition. This finding gives evidence that these parasites may have some effect on these animals and affect populations both *in situ* and *ex situ*. The capybara did not share any common gastrointestinal cestodes with the agouti or the lappe. This could be due to the large difference in size as well as differences in feeding behavior. With the capybara being a semi-aquatic herbivore and the agouti and lappe considered as scatter hoarding frugivores (32).

**Table 1:** Prevalence of adult cestodes of hystricomorphic rodents and various geographical locations.

**References**	**Host**	**Cestode**	**Prevalence, %** **(x/y)**	**Geographic location**	**Method of identification**
Suepaul et al. (14)	*D. leporina*	Unidentified cestode	7.69 (1/13)	Trinidad	Faecal flotation
Matamoros et al. (15)	*C. paca*	*Taenia* spp.	2.14 (3/140)	Costa Rica	Faecal flotation
Uribe et al. (17)	*H. hydrochaeris*	*Monoecocestus* spp.	6.5 (3/46)	Orinoco Basin, Colombia	Centrifugal flotation
Sinkoc et al. (18)	*H. hydrochaeris*	*M. hydrochoeri* *M. jacobi*	50 (12/24) 41.67 (10/24)	Rio Grande, Brazil	Morphology of adults
Souza et al. (19)	*H. hydrochaeris*	*M. macrobursatus* *M. hydrochaeris* *Monecocestus* spp. Anoplocephalidae	50 (5/10) 70 (7/10) 10 (1/10) 10 (1/10)	Upper Parana, Brazil	Morphology of adults
Wendt et al. (20)	*H. hydrochaeris*	*M. hagmanni* *M. macrobursatum*	17.64 (6/34) 2.94 (1/34)	Rio Grande do Sul, Brazil	Morphology of adults
Corriale et al. (21)	*H. hydrochaeris*	*M. hydrochoeri*	11.06 (22/200)	Corrientes, Agentina	Faecal floatation
El-Kouba et al. (22)	*H. hydrochaeris*	Unidentified cestode	30.3 (10/33)	Parana, Brazil	Faecal floatation
Casas et al. (23)	*H. hydrochaeris*	*M. hagmanni* *M. macrobursatum* *M. hydrochoeri*	12 (5/41) 34 (14/41) 12 (5/41)	Bolivia	Morphology of adults
Costa et al. (24)	*H. hydrochaeris*	*M. hydrochoeri* *M. hagmanni*	56.5 (12/23) 78.2 (18/23)	Pantanal Sul Mato Grossense, Brazil	Morphology of adults
Salas and Herrera (25)	*H. hydrochaeris*	*M. macrobursatum* *M. hagmanni*	97.5 (39/40) 72.5 (29/40)	Apure State, Venezuela	Morphology of adults
Sinkoc et al. (26)	*H. hydrochaeris*	*M. hydrochoeri* *M. hagmanni*	28.57 (2/7) 42.86 (3/7)	São Paulo, Brazil	Morphology of adults
Bonuti et al. (27)	*H. hydrochaeris*	*M. macrobursatum* *M. hagmanni* *M. hydrochoeri* *Monoecocestus* (immature form)	56.66 (17/30) 23.33 (7/30) 23.33 (7/30) 46.66 (14/30)	Panatanal do Mato Grosso do Sul, Brazil	Morphology of adults
Foster et al. (28)	*L. maximus*	*Monoecocestus* spp.	23.67 (9/38)	Argentina	Morphology of adults
Martino et al. (29)	*M. coypus*	*Taenia* spp. *R. avetjanae* *Anoploceplala* spp. *H. octocoronata*	3.7 (4/108) 0.9 (1/108) 0.9 (1/108) 1.8 (2/108)	Argentina	Morphology of adults
Benati et al. (30)	*M. coypus*	*H. octocoronata*	100 (3/3)	Brazil	Morphology of adults
d'Ovidio et al. (31)	*C. chinchilla*	*H. nana*	25 (6/24)	Italy	Faecal floatation

Identification of these parasites can be done through fecal flotation techniques as well as through the morphology of adult worms (10). Several authors have investigated gastrointestinal parasites of the agouti (33–36), lappe (37) and capybara (38, 39) using fecal flotation techniques without identifying any cestodes. Several cestodes have been identified in the gastrointestinal tract of the nutria. In most cases these parasites did not cause any clinical illness. Some of the parasites identified were: *Anaplocephala* spp. Goeze, 1782, *Taenia* spp. Goeze, 1782, *Hymenolepsis avetjanae* Ransom, 1901, *Hymeolepsis octocoronata* Ransom, 1901 (29, 30, 40). In the nutria, these parasites had varied prevalence based on location. However, the prevalence was relatively low. In the guinea pig, *Monoecocestus parcitesticulatus* Rego, 1960 was found in Brazil (41). While, *Monecocestus* spp. was reported in the plains vizcacha in Argentina (28). In chinchillas, *Hymenolepis nana* Ransom, 1901 was present in 25% of the animals sampled in Italy (31).

## Cestodes in Body Tissues

Cestodes found in body tissues of the agouti and the lappe are *Echinococcus vogeli* and *Echinococcus oligarthrus* (42–48). These parasites are usually found as metacestodes in the liver, subcutaneous tissue and the heart. The agouti and the lappe are intermediate hosts for these parasites (49, 50), with the definitive host being neo-tropical canids (wild and domestic) and felids (pumas, jaguars, jaguarandis and ocelot) (51–54). The lappe (*C. paca*) is frequently seen with *E. vogeli* in the liver, grossly having a polycystic appearance (43, 46). However, *E. oligarthrus* identified in the agouti was found in the subcutaneous region and the heart (45, 48). Lesions from *E. oligarthrus* appear to be unicystic in appearance (45). The cestodes have a neo-tropical geographical distribution affecting animals in Bolivia, Peru, Brazil and Columbia (see Table 2).

**Table 2:** Prevalence of immature cestodes of hystricomorphic rodents and various geographical locations.

**References**	**Host**	**Cestode**	**Prevalence %** **(x/y)**	**Geographical location**	**Method of identification**
Morales et al. (48)	*C. paca*	*E. vogeli*	46.2 (44/93)	Columbia	Histopathology
	*D. leporine*		0 (0/20)		
	*H. hydrochaeris*		0 (0/57)		
	*Proechymis* spp.		0.27 (1/369)		
Mayor et al. (42)	*C. paca*	*E. vogeli*	11.7 (15/128)	Peru	Histopathology
Almeida et al. (44)	*C. paca*	*E. vogeli*	60 (3/5)	Brazil	Histopathology
Umhang et al. (55)	*M. coypus*	*E. multilocularis* *T. taeniaformis* *T. polyacanta* *T. mustelae* *T. martins*	0.4 (2/531) 1.3 (8/531) 0.9 (5/531) 1.1 (6/531) 0.2 (1/531)	Switzerland	PCR and Gene Sequencing

In the agouti (*D. leporina*), there have been clinical reports of *E. oligarthrus* in from wild caught animals from Guyana and Brazil (45). These animals had body weights ranging from 3.02 to 3.44 kg and appeared visibly healthy. However, these animals had subcutaneous cysts ranging from 0.5 to 2.0 cm. *E. oligarthrus* was confirmed using ultrasonography, radiography and histology (45). In these cases, praziquantel and albendazole were given but no significant change was seen in the size of the cysts but no new cysts were seen developing.

In the lappe (*C. paca*), *E. vogeli* have been identified in Peru (42, 56), Bolivia (47), Brazil (44) and Colombia (48). This parasite was found in the liver of infected lappe and confirmation was made through histological techniques. In Columbia, 44 of 93 lappe were infected with *E. vogeli*. Surprisingly, no agoutis (out of 0/20) and capybara (0/57) were infected with *E. vogeli* in the liver (48). However, some hunters did provide information of hydatid cysts present in the heart, muscle and liver of the agouti (*D. fulginosa*) (48). Similarly, hydatid cysts were found in the liver of 60% of lappe sampled in Brazil (44) and 11.7% in Peru (42). It is important to highlight that *E. vogeli* and *E. oligarthrus* were not identified in the capybara (*H. hydrochaeris*). The reason for this absence of this parasite in the capybara can be due to its feeding habits as well as its ecological role. In comparison to the agouti and the lappe, capybaras are semi- aquatic herbivores and much larger than the two rodents mentioned above. Also, there is limited contact between the predators (wild canids and felids) of the agouti and lappe as compared to the capybara.

The nutria has been reported as an intermediate host for *E. multilocularis* in endemic areas (55, 57). In the nutria, lesions were found in the liver. Umhang et al. (55) utilized molecular techniques in the identification of metacestodes. Several metacestodes were identified which included: *E. multilocularis, T. taeniformis, T. polyacantha, T. mustelae*, and *T. martins*. It must be noted that the prevalence of these parasites were quite small ranging from 0.2 to 1.3%. In the chinchilla, *T. crassiceps* were found in several tissues and confirmed using molecular techniques (58). *H. nana*, which is a zoonotic cestode was also identified in the liver of the chinchilla (59).

## Cestodes of Zoonotic Importance

Humans can become infected with *E. vogeli* and *E. oligarthrus* when they consume eggs which have been passed from the feces of the definitive host. The feeding of hunting dogs viscera of neo-tropical rodent (agouti and lappe) allows the dog to become the definitive host. Consumption of eggs can occur through contamination of food and water with the feces of the dog (60). This disease is usually seen affecting persons in the rural neo-tropics that have contact with wild species (60, 61). *E. vogeli* is more prevalent than *E. oligarthrus*, with cystic lesion forming primarily in the liver but can also be found in other abdominal organs (mesenteries, spleen, and uterus) and the lungs (62–70). *E. oligarthrus* has been found in the eye as well as the heart (56, 70, 71). In recent times *E. oligarthrus* has also been identified in the liver using molecular techniques. Similarly, *E. vogeli* has been noted to occur in the mesenteries without liver involvement (72).

Neotropical echinococcosis is diagnosed through demonstration of polycystic masses in the abdomen, radiographic imaging, patients' history, serological tests, and parasitological diagnosis based on histology (60). Molecular tools have been used in the identification of *E. vogeli* and *E. oligarthrus* (73, 74). These new tools are more accurate than the morphological techniques or gross lesions of the affected organ in the identification of the two species of neo-tropical echinococcosis. Serological tests have been used but they appear to be inaccurate. In cases where the cysts are calcified serological tests may be negative. Also, serology cannot be used to differentiate the species of *Echinococcus* that is present within the patient (75).

Treatment of this disease usually involves the use of anti-parasitic drugs in conjunction with surgery. In most cases albendazole is used for a prolonged period (3 to 6 months) with the removal of cysts from affected organs (76). Some reports have also transplanted liver tissue that was affected in conjunction with medical anti-parasitic treatment (77). Some studies (*in vitro* and *vivo*) have been done to understand the proliferation of the metacestodes (78, 79). Within the normal intermediate proliferation is restricted to the liver, however, when infection occurs in an abnormal intermediate host there is proliferation to other organs within the abdomen (79). Preventive measures that can be employed to reduce the incidence of this disease in humans is: (1) not to feed hunting dogs viscera of neo-tropical rodents (lappe and agouti), (2) regular deworming of dogs with benzimidazoles (e.g., mebendazole, fenbendazole, albendazole), (3) proper sanitary measures after interaction with pets, (4) washing of fruits and vegetables before consumption.

## Conclusion

This review showed that capybara had the greatest quantity of research done with respect to gastrointestinal cestodes. The prevalence of these parasites varied in the capybara with respect to location. In contrast, there was little work that reported gastrointestinal cestodes in the other neo-tropical rodent. The metacestodes (immature cestodes) were only found in the tissue of the lappe, spiny rat, chinchilla, nutria and the agouti. These immature forms were usually found in the liver, lungs, muscles and other abdominal organs. The capybara was found to negative for these metacestodes which shows the difference in the feeding behavior of the capybara as compared to the lappe and the agouti. The lappe and the agouti serve as intermediate hosts of *E. vogeli* and *E. oligarthrus* which have public health implications to humans. The nutria can serve as an intermediate host for *E. multilocularis*.

## Recommendations

Future work should focus on investigating cestodes found in the body tissue of neo-tropical rodents using molecular technologies. This will give an accurate prevalence of the specific parasites that are of public health concern that utilize these animals as intermediate hosts. Investigations can also be done on the effect gastrointestinal cestodes have on the health and performance of neo-tropical rodents with the potential to be domesticated.

## Author Contributions

The author confirms being the sole contributor of this work and has approved it for publication.

## Conflict of Interest

The author declares that the research was conducted in the absence of any commercial or financial relationships that could be construed as a potential conflict of interest.

## Publisher's Note

All claims expressed in this article are solely those of the authors and do not necessarily represent those of their affiliated organizations, or those of the publisher, the editors and the reviewers. Any product that may be evaluated in this article, or claim that may be made by its manufacturer, is not guaranteed or endorsed by the publisher.
